# Validation of the Saga Fall Injury Risk Model

**DOI:** 10.7150/ijms.92837

**Published:** 2024-05-19

**Authors:** Risa Hirata, Naoko E. Katsuki, Shizuka Yaita, Eiji Nakatani, Hitomi Shimada, Yoshimasa Oda, Midori Tokushima, Hidetoshi Aihara, Motoshi Fujiwara, Masaki Tago

**Affiliations:** 1 Department of General Medicine, Saga University Hospital, Saga, Japan.; 2 Graduate School of Public Health, Shizuoka Graduate University of Public Health, Shizuoka, Japan.; 3 Shimada Hospital of Medical Corporation Chouseikai, Saga, Japan.; 4 Department of General Medicine, Yuai-Kai Foundation and Oda Hospital, Saga, Japan.

**Keywords:** Accidental Falls, Accidental Injuries, Validation Studies, Logistic Models, Risk Factors

## Abstract

**Background:** Predicting fall injuries can mitigate the sequelae of falls and potentially utilize medical resources effectively. This study aimed to externally validate the accuracy of the Saga Fall Injury Risk Model (SFIRM), consisting of six factors including age, sex, emergency transport, medical referral letter, Bedriddenness Rank, and history of falls, assessed upon admission.

**Methods:** This was a two-center, prospective, observational study. We included inpatients aged 20 years or older in two hospitals, an acute and a chronic care hospital, from October 2018 to September 2019. The predictive performance of the model was evaluated by calculating the area under the curve (AUC), 95% confidence interval (CI), and shrinkage coefficient of the entire study population. The minimum sample size of this study was 2,235 cases.

**Results:** A total of 3,549 patients, with a median age of 78 years, were included in the analysis, and men accounted for 47.9% of all the patients. Among these, 35 (0.99%) had fall injuries. The performance of the SFIRM, as measured by the AUC, was 0.721 (95% CI: 0.662-0.781). The observed fall incidence closely aligned with the predicted incidence calculated using the SFIRM, with a shrinkage coefficient of 0.867.

**Conclusions:** The external validation of the SFIRM in this two-center, prospective study showed good discrimination and calibration. This model can be easily applied upon admission and is valuable for fall injury prediction.

## Introduction

Falls in hospitals pose a significant problem because of their potential to cause physical burden 1 and psychological distress, such as fear and anxiety, among patients, their families, and healthcare workers 2,3. Various prediction models have been developed to prevent falls; however, injuries occur in 15.7%-36.9% of all falls, with severe injuries occurring in 2.2%-12.8% of cases 4,5. Fall-related injuries increase healthcare costs 6. Many hospitals routinely use bed alarms, low beds, hip protectors, and other medical resources to prevent falls and fall injuries 7,8. In addition to these physical preventative measures, fall prevention education and awareness for patients at high risk of falling and their families can reduce falls and associated disabilities 9. However, given the limited medical resources and personnel, to ensure high-quality safety, it is possible to reduce medical costs by focusing on patients at a higher risk of fall injuries and efficiently allocating resources rather than targeting all patients.

Several models have been developed to predict fall-related injuries in older adults 10,11. However, no specific established models or studies have targeted adult inpatients. Therefore, in our previous study, we developed a fall injury prediction model (Saga Fall Injury Risk Model [SFIRM]), using data from those with fall injuries and those without fall injuries or falls in the hospital that could be used to predict fall injuries 12. The SFIRM is not a model for assessing the presence or absence of falls, but a simple model that can predict whether a fall injury will occur during hospitalization by evaluating all inpatients only once on admission 12. The SFIRM comprises six factors including age, sex, emergency transport, medical referral letters, Bedriddenness Rank, and history of falls (Figure S1) 12. In previous studies, Bedriddenness Rank has shown good inter-rater reliability and criterion-related validity 13. Bedriddenness ranks were categorized into five major classes (normal, J: independence/autonomy, A: housebound, B: chair-bound, and C: bed-bound) 13. Although external validation was not conducted in the previous study, internal validation showed an area under the curve (AUC) of 0.794 (95% confidence interval [95% CI]: 0.762-0.826) for this model 12. Furthermore, the SFIRM can be applied upon admission and was developed using factors associated with fall injuries 12. Among the six constituent factors, the odds ratios for Bedriddenness Rank were remarkably higher than those for other factors 12. In addition, age 14,15, sex 16, history of falls 17, and Bedriddenness Rank A 18 are known risk factors for fall injuries. In contrast, the absence of emergency transport, presence of a medical referral letter, and Bedriddenness Ranks J, B, and C were newly identified predictive factors for fall injuries. However, after our prior research, the associations between these new predictive factors and fall injuries have not been adequately verified, and the reproducibility of this finding has not been confirmed.

This prospective study conducted in two hospitals, an acute and a chronic care hospital, aimed to externally validate the SFIRM. Furthermore, we aimed to verify whether the constituent factors of the model are reproducible for fall injuries.

## Methods

### Study design

This was a two-center, prospective, observational study. We recruited inpatients aged 20 years or older, admitted to two hospitals with different backgrounds, namely Yuai-Kai Foundation and Oda Hospital (an acute care hospital) and Saga City Fuji-Yamato Spa Hospital (provides both acute and chronic care), from October 2018 to September 2019 (Table S1). This study targeted patients of the same age group as in the previous study 12.

### Data collection and definitions

All data, including admission date, patient age at admission 14,15, sex 12,16, department 19, presence of emergency transport 12, presence of a medical referral letter written by a primary physician outside of the two hospitals who saw a patient before admission 12, the Ministry of Health, Labour and Welfare's Activities of Daily Living scale (Bedriddenness Rank and Cognitive Function Score) 12, Barthel Index, Katz Index, Mini-Mental State Examination (MMSE) 20, ABC-dementia scale 21, use of hypnotic medication 22, presence of permanent residual damage from previous stroke 23, history of falls 12, visual impairment 18, surgery during hospital stay 24,25, rehabilitation 26, occurrence of in-hospital falls, and discharge date, were extracted from the electronic medical records of each hospital. We collected the data of factors related to falls or fall injuries. Detailed definitions of each item are provided in the Supplement 1. The number of falls, which were documented when the falls were reported by the patient or discovered by the attending nurse, was aggregated from incident and accident reports. Falls were defined as unexpected falls from any height or position regardless of injury, such as falls on stairs, chairs, or beds while standing, walking, sitting, or in a supine position. Fall-related injuries were defined as falls resulting in severity 1 or higher, according to the “Reasons for Falls and Falls and Injury Severity Input Criteria” developed from the Maryland Hospital Association Center for Performance Sciences' Quality Indicator Project 12,27. Severity 1 indicates that although an injury has occurred, there are no sequelae or extension of hospital stay. We divided the patients into two groups: the “with fall injury” group, which included those who fell and had an injury with a severity of 1 or higher, and the “without fall injury” group, which included both those who did not fall and those who fell but did not have any fall injuries. We judged that all those who suffered injuries related to falls were considered to have fall injuries.

### Assessment and prevention of falls during the study period

The predictive results generated from the SFIRM were not disclosed to attending nurses or physicians. Instead, the usual assessment of fall risk for hospitalized patients was implemented using each hospital's proprietary assessment tools and subsequent fall prevention measures based on those assessments. Depending on the patient's risk and circumstances, interventions such as guidance on appropriate footwear; use of sensors, cameras, impact-absorbing mats, low-height beds, belts, and bed rails; and assistance with toilet transfers were provided.

### Statistical analysis

Statistical analysis of the survey variables was performed for the entire group, the group with fall injuries, and the group without fall injuries. Continuous and categorical variables were presented as median values (interquartile range) and absolute numbers (percentages), respectively. The standardized mean differences of each patient were analyzed using EZR (Saitama Medical Center, Jichi Medical University, Saitama, Japan), a graphical user interface for R (The R Foundation for Statistical Computing, Vienna, Austria) 28. More precisely, it is a modified version of R commander designed to add statistical functions frequently used in biostatistics 28. This exploratory analysis did not consider multiplicity. We evaluated the predictive performance of the SFIRM by calculating the AUC, 95% CI, and shrinkage coefficient for all patients. In addition, for outcomes related to fall injuries, univariate analysis was conducted for variables that showed significance, and potential confounders such as age and sex were included as covariables in a logistic regression analysis. Analyses were performed using IBM SPSS Statistics, version 25 (IBM Corp., Armonk, NY, USA), with the significance level set at p less than 0.05.

### Ethical considerations

This study adhered to the “Ethical Guidelines for Medical and Health Research Involving Human Subjects” provided by the Ministry of Health, Labour and Welfare and the Ministry of Education, Culture, Sports, Science and Technology in Japan. Ethical approval for this research was obtained from the Ethics Committee of Yuai-Kai Foundation and Oda Hospital (Approval No. 20230904). Informed consent was obtained from all patients using a comprehensive hospital agreement method, and patient anonymity was ensured.

### Sample size

We calculated a minimum sample size of 2,235 patients for this study based on an effect size of 0.20 (predicted AUC of 0.70 and null hypothesis AUC of 0.50), a fall injury rate of 0.7%, an alpha error of 0.05, and a beta error of 0.20, using an estimated AUC of 0.794, as reported in a previous study 12.

## Results

Of the 3,647 cases admitted during the study period, we excluded 96 cases with suspected input errors (e.g., Bedriddenness Rank of normal but Barthel Index < 10, Cognitive Function Score of normal but MMSE was evaluated, Cognitive Function Score of normal but ABC-dementia scale score < 10), and 34 cases with missing data, resulting in a total of 3,549 cases for analysis (Figure S2). The median age of the entire population was 78 years, and the proportion of men was 47.9%. The median length of hospital stay was 10 days. Among the cases, 35 (0.99%) had fall injuries. In the group with fall injuries, the median age was 86 years, 37.1% were men, and the median length of hospital stay was 37 days. The 34 cases with missing data had a median age of 60 years, 61.8% were men, 73.5% were scheduled for hospitalization, 41.2% had surgery, 2.9% had rehabilitation, and the median length of stay was 5 days.

The results of the univariate analysis are presented in Table 1. Age was significantly higher (86 years vs. 78 years) and the length of hospital stay was significantly longer (37 days vs. 9 days) in the group with fall injuries than in the group without fall injuries. Regarding admission type, emergency admissions (80% vs. 38.3%), the use of hypnotic medications (25.7% vs. 9.7%), history of falls (34.3% vs. 9.3%), visual impairment (60.7% vs. 34.0%), and rehabilitation (65.7% vs. 36.6%) were significantly more common in the fall injury group than in the without fall injury group. In contrast, the incidence of surgery was significantly lower in the fall injury group than in the without fall injury group (11.4% vs. 28.1%). Higher proportions of fall injuries were observed in the groups with Bedriddenness Ranks J, B, and C, as well as in the groups with Cognitive Function Scores I to IV, and these distributions differed significantly between the fall injury and without fall injury groups. The Barthel Index (55 vs. 100) and ABC-dementia scale (85 vs. 117) scores were significantly lower in the group with fall injuries than in the group without fall injuries. No significant differences were observed between the fall and without fall injury groups regarding sex, emergency transport, medical referral letters, permanent residual damage from previous stroke, or MMSE scores.

**Figure 1:**
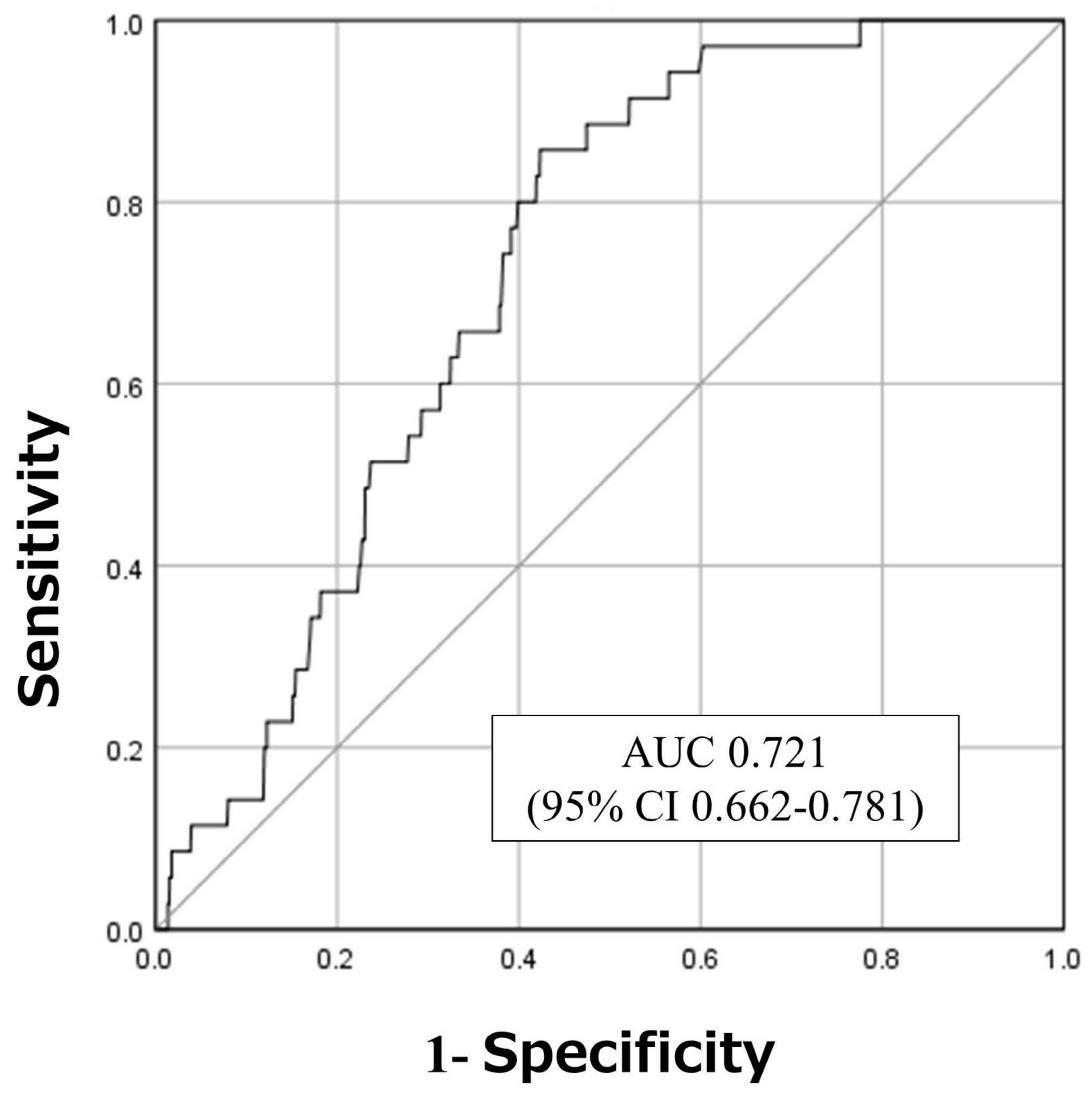
Receiver operating characteristics and areas under the curves. AUC: area under the curve; 95% CI: 95% confidence interval.

**Figure 2:**
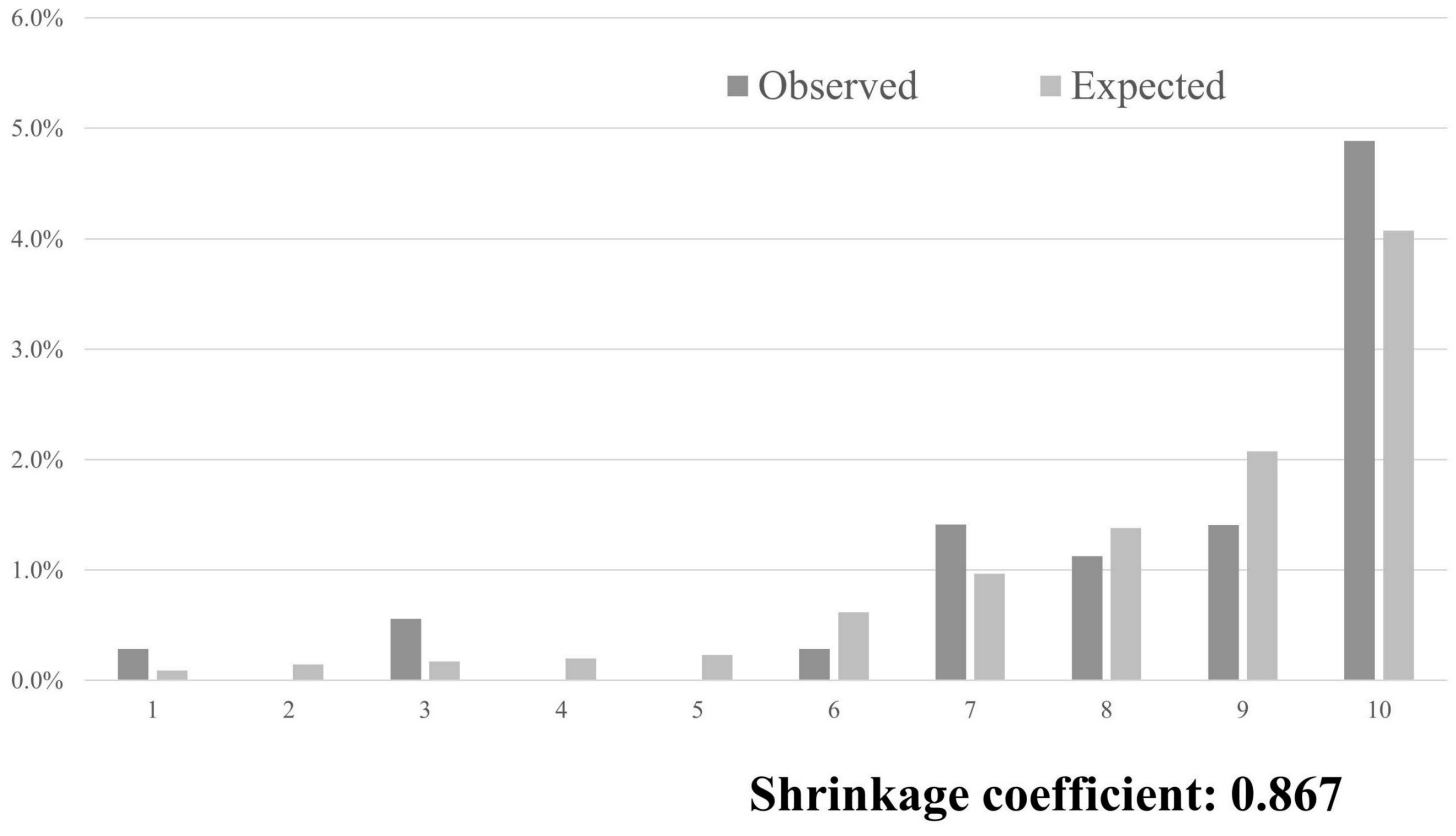
Predicted and observed rates of falls in 10 groups, divided into 10 deciles by score using the predictive model.

The AUC, which was used to assess the performance of the SFIRM, was 0.721 (95% CI: 0.662-0.781; Figure 1). The observed incidence of falls was consistent with the predicted incidence calculated using the SFIRM, with a shrinkage coefficient of 0.867 (Figure 2). The cutoff values for the model with 91% sensitivity and 48% specificity points was -5.80, and with the 14% sensitivity and 90% specificity points was -3.73. The cutoff value according to Youden's Index was -5.09, with sensitivity of 86% and specificity of 58% (Table 2).

Variables with significance in the univariate analysis, including age, history of falls, Bedriddenness Rank, and sex, were entered as covariables in a forced logistic regression analysis. Age (p = 0.958), sex (p = 0.488), and Bedriddenness Rank A (p = 0.075) were not significantly associated with fall injuries. Bedriddenness Ranks J, B, and C had odds ratios of 8.0 (95% CI: 1.71-37.07, p = 0.008), 13.5 (95% CI: 3.36-54.69, p < 0.001), and 6.1 (95% CI: 1.38-27.44, p = 0.017), respectively, all of which were significantly associated with fall injuries (Table 3).

## Discussion

This study prospectively validated the SFIRM that was developed in a previous study on in-hospital fall injuries among inpatients 12. The SFIRM showed good discrimination, with an AUC of 0.721, and both calibration and discrimination were satisfactory. Furthermore, the results of the logistic regression analysis demonstrated a significant association between fall history and Bedriddenness Ranks J, B, and C.

In contrast to the previous development study, the present study conducted an external validation using prospectively collected data. The SFIRM utilizes data from an acute care hospital where the model was initially developed and included data from a different background, namely, a chronic care hospital. Despite this diverse setting, the model demonstrated good accuracy, which was similar to that of the previous study 12, confirming the utility of our SFIRM. Furthermore, the SFIRM can be applied at the time of admission without requiring special additional tests such as blood examinations or physical performance assessments because it is simple and comprises only six factors 12. Moreover, this model incorporated the Bedriddenness Ranks, which is widely used in Japanese healthcare and long-term care settings 29,30. By identifying patients at risk of fall injuries using the convenient and valuable SFIRM, severe fall injuries that tend to result in high inpatient medical expenses can be prevented 31, thereby contributing to cost containment. Therefore, the SFIRM is a socially beneficial and suitable fall injury prediction model for healthcare and long-term care settings.

Among the six factors of the SFIRM, the univariate analysis showed that age, history of falls, and Bedriddenness Rank were significantly different between the fall and without fall injury groups. In the logistic regression analysis, history of falls and Bedriddenness Ranks J, B, and C showed significant associations with fall injuries. These factors may be useful factors for predicting fall injuries. History of falls is widely recognized as a contributing factor, and previous studies have reported that patients with history of a fall are at a higher risk of sustaining injuries during subsequent falls 17. Bedriddenness Rank is a well-established activities of daily living scale used in Japanese long-term care and healthcare settings, showing good criterion-related validity with existing measures such as the Barthel Index and Katz Index 13. Bedriddenness Rank J represents individuals with some form of impairment but are mostly independent in their daily lives 18. Consequently, factors such as reduced balance capabilities due to physical impairments 32 and decreased muscle strength 33 might contribute to increased risk of fall injuries in such individuals compared with those in healthy individuals. In addition, cognitive impairment may lead to delirium 34 upon admission, thereby increasing the risk of falls 35. Factors associated with fall injuries, such as lower limb muscle weakness and the presence of caregivers, have been reported 5, and the judgment criteria for Bedriddenness Rank B include the necessity of indoor assistance 18, which may also contribute to fall injuries. Furthermore, the criterion for Bedriddenness Rank C, that is, whether the patient can turn over in bed independently 18, is predicted to be associated with physical function decline and muscle weakness. Given the significant relationship between physical function decline and injuries, such as fractures or trauma resulting from falls 10, a potential association with fall injuries may exist. In addition, patients who are bedridden are at higher risk of osteoporosis, which increases the risk of fractures 36, potentially contributing to fall injuries.

### Limitations

This study was an external validation of SFIRM developed in previous research. Therefore, it was necessary to evaluate the population with the same condition as in the previous study, and the validation was performed in groups with fall injuries and without fall or fall injuries. This was a prospective study; however, owing to the limited number of fall injury events, we could not conduct a multivariable analysis with sufficient covariables. In addition, no adjustments for interventions aimed at preventing falls were made. Additionally, there could be fall or fall injury risk factors that were not collected in this study; therefore, we could not adjust for all confounding factors. Furthermore, we could not evaluate the inter-rater reliability for each item. The group with missing data may have included more younger patients with short-term planned hospitalizations than did the group analyzed in the present study, which could have potentially influenced the results.

## Conclusion

The external validation of the SFIRM, which was prospectively conducted in two hospitals in the population with the same condition as in the previous study, showed good discrimination and calibration in this study. The SFIRM can be easily applied upon admission and is valuable for fall injury prediction. Moreover, history of a fall and Bedriddenness Rank were associated with fall injuries, potentially contributing to the prediction of such injuries. Therefore, further studies validating the model in a larger or a different population are necessary.

## Supplementary Material

Supplementary figures, table and information.

## Figures and Tables

**Table 1:** Characteristics of patients and results of univariable analysis.

Variable, Category	All patients n = 3549	With Fall injury	Without Fall injury	SMD
		n = 35	n = 3514	
Age, years	78 (65-87)	86 (78-90)	78 (65-87)	0.602
Sex, Men	1700 (47.9)	13 (37.1)	1687 (48.0)	0.221
Department, Internal Medicine	1998 (56.3)	27 (77.1)	1971 (56.1)	0.584
Department, Neurosurgery	68 (1.9)	2 (5.7)	66 (1.9)	
Emergency admission, Yes	1372 (38.7)	28 (80.0)	1344 (38.3)	0.938
Transported by ambulance, Yes	474 (13.4)	6 (17.1)	468 (13.3)	0.107
Referral letter, Presence	1065 (30.0)	7 (20.0)	1058 (30.1)	0.234
Hypnotic medications, Using	344 (9.9)	9 (25.7)	335 (9.7)	0.428
Permanent residual damage from previous stroke, Presence	218 (6.3)	3 (8.6)	215 (6.3)	0.088
History of falls, Presence	338 (9.5)	12 (34.3)	326 (9.3)	0.629
Visual impairment, Presence	1135 (34.2)	17 (60.7)	1118 (34.0)	0.555
Bedriddenness rank,^a^ Normal	1788 (50.4)	3 (8.6)	1785 (50.8)	1.134
Bedriddenness rank,^a^ J	317 (8.9)	5 (14.3)	312 (8.9)	
Bedriddenness rank,^a^ A	410 (11.6)	4 (11.4)	406 (11.6)	
Bedriddenness rank,^a^ B	517 (14.6)	16 (45.7)	501 (14.3)	
Bedriddenness rank,^a^ C	517 (14.6)	7 (20.0)	510 (14.5)	
Cognitive function score,^b^ Normal	2185 (61.8)	5 (14.7)	2180 (62.2)	1.150
Cognitive function score,^b^ I	423 (12.0)	8 (23.5)	415 (11.8)	
Cognitive function score,^b^ II	341 (9.6)	10 (29.4)	331 (9.4)	
Cognitive function score,^b^ III	453 (12.8)	8 (23.5)	445 (12.7)	
Cognitive function score,^b^ IV	119 (3.4)	3 (8.8)	116 (3.3)	
Cognitive function score,^b^ M	16 (0.5)	0 (0.0)	16 (0.5)	
Barthel index	100 (52-100)	55 (25-75)	100 (55-100)	0.680
ABC-dementia Scale	117 (91-117)	85 (68-106)	117 (91-117)	0.580
Mini-Mental State Examination^c^	13 (0-21)	16 (9-20)	13 (0-21)	0.224
Surgery, Undergone	991 (27.9)	4 (11.4)	987 (28.1)	0.428
Rehabilitation, Undergone	1309 (36.9)	23 (65.7)	1286 (36.6)	0.609
Length of hospital stay (days)	10 (5-18)	37 (22-61)	9 (5-18)	1.103

Continuous and categorical variables are shown as median (interquartile range) and frequency (percent). SMD, standardized mean difference. ^a^ Bedriddenness Ranks: J, independence/autonomy; A, house-bound; B, chair-bound; C, bed-bound. ^b^ Cognitive Function Scores: I, almost independent in daily living with only slight cognitive impairment; II, independent with slight difficulty in daily living or communication under careful overseeing; III, dependent in daily living or communication; IV, dependent in daily living or communication, and requires constant care; M, severe psychological symptoms, troubled behaviors or severe physical disorders requiring specialized medical service. ^c^ Mini-Mental State Examination was assessed within 72 h of admission, for those determined to have an abnormal Cognitive Function Score.

**Table 2:** Validation of the predictive model with the cutoff points determined in the present study.

Statistics for 3 cutoff points	Overall	Overall With the cutoff points set in the development study
Cutoff value for scores	-5.80	-5.27
Probability ^a^	0.3	0.5
Sensitivity	91	86
Specificity	48	55
Positive predictive value	1.7	1.9
Negative predictive value	99.8	99.7
Cutoff value for scores	-5.09	-5.08
Probability ^a^	0.6	0.6
Sensitivity	86	83
Specificity	58	58
Positive predictive value	2.0	1.9
Negative predictive value	99.8	99.7
Cutoff value for scores	-3.73	-3.80
Probability ^a^	2.3	2.2
Sensitivity	14	14
Specificity	90	88
Positive predictive value	1.4	1.2
Negative predictive value	99	99

^a^ The value was calculated as the probability of a fall for patients with defined score. Probability=100×Exp(score)/(1+Exp(score))

**Table 3:** Results of multivariable logistic regression analysis.

Variable, Category (Reference)	OR	95% CI	p value^a^
Age	1.0	0.97-1.03	0.958
Sex, Female (Male)	1.3	0.63-2.60	0.488
History of falls, Presence (Absence)	2.8	1.32-5.72	0.007
Bedriddenness rank, J (Normal)	8.0	1.71-37.07	0.008
Bedriddenness rank, A (Normal)	4.4	0.86-22.00	0.075
Bedriddenness rank, B (Normal)	13.5	3.36-54.69	< 0.001
Bedriddenness rank, C (Normal)	6.1	1.38-27.44	0.017

OR: odds ratio; 95% CI: 95% confidence interval ^a^ p values for Wald test.

## Abbreviations

AUC: area under the curve
CI: confidence interval
MMSE: Mini-Mental State Examination
SFIRM: Saga Fall Injury Risk Model
