# Supplementary material for: Validation of the Saga Fall Injury Risk Model
Source: Int J Med Sci. 2024 May 19;21(8):1378–84. doi: 10.7150/ijms.92837 (PMC11186423; doi:10.7150/ijms.92837)
Supplement: Supplementary file 1 — Supplementary figures, table and information. [file ijmsv21p1378s1.pdf]

Supplementary Material

Figure S1. Formula of the Saga Fall Injury Risk Model.

Saga Fall Injury Risk Model

Score ＝−8.672 + 0.034 × (age) + (sex: female= －0.641) + (emergency transport: presence= －0.887, missing: －0.551)+ (medical referral letter; presence = 0.399, missing = 1.189) + (Bedriddenness ranks; J = 1.597, A = 2.188, B = 1.986, C = 1.510, others = 0.154) + (history of falls = 0.542)

Figure S2. Data flow diagram.

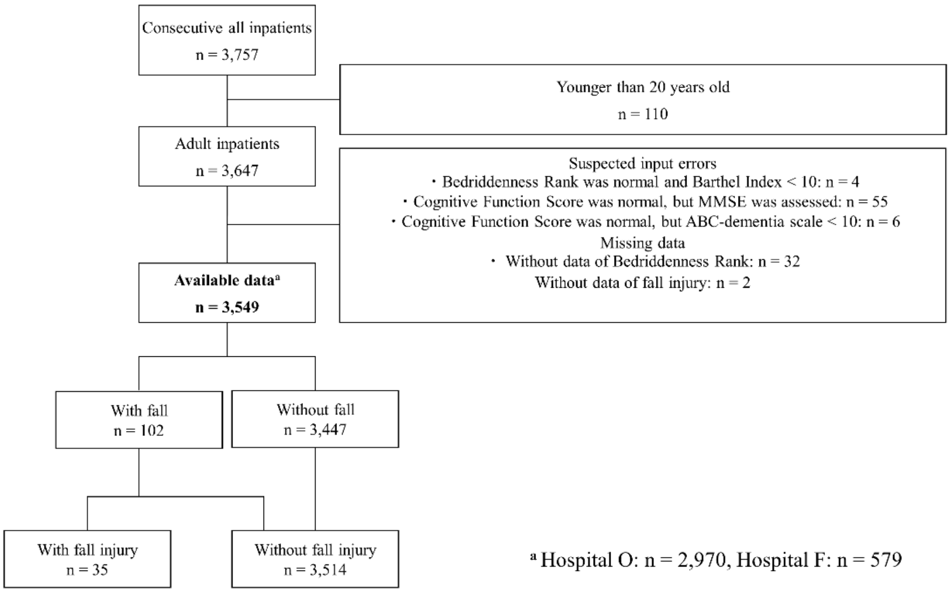

MMSE: Mini-Mental State Examination

Table S1. Characteristics of each hospital.

	Population of the Local Government Area (inhabitants)	Hospital Function	Number of Medical Departments	Number of Beds	Annual Number of Inpatients	Average Length of Hospital Stay (days)
O hospital	28,000	Acute	11	111	3,100	12.1
F hospital	230,000	Acute and Chronic	5	98 (54 beds for acute care)	650	19.1 (Overall), 71.3 (Chronic care)

Supplement 1. Definition and collection methods of the items.

The admitting departments were categorized as internal medicine, neurosurgery, and other. Functional status, as measured by Bedriddenness Rank, Cognitive Function Score, Barthel Index, Katz Index, hypnotic medication, presence of permanent residual damage from previous stroke, history of falls, and visual impairment for all inpatients aged 20 years or older were assessed by the attending nurses within 72 hours of admission. The Bedriddenness Rank is a public scale developed by the Ministry of Health, Labour, and Welfare in Japan. Bedriddenness Rank and Cognitive Function Score have been confirmed to have good inter-rater reliability and criterion-related validity. It includes five categories in the primary classification and nine in the detailed classification, whereas the Cognitive Function Score includes six categories in the primary classification and eight in the detailed classification. In this study, we utilized the primary classification with five categories for Bedriddenness Rank (normal, J: independence/autonomy, A: housebound, B: chair-bound, or C: bed-bound) and the primary classification with six categories for Cognitive Function Scores (normal, 1, 2, 3, 4, and M). The MMSE and ABC-dementia scales were assessed by the attending nurse or research assistant within 72 hours of admission, for patients whose Cognitive Function Scores were abnormal based on the initial assessment by the attending nurse at admission.

Hypnotic medications included benzodiazepines and non-benzodiazepines, which were defined according to their definition in previous studies, excluding melatonin receptor agonists and orexin receptor antagonists. Permanent residual damage from a previous stroke was defined as lower limb paralysis, regardless of the severity, resulting from cerebral infarction, cerebral hemorrhage, or subarachnoid hemorrhage. History of falls within the past year before the study was considered positive, regardless of the trauma or location. Visual impairment was defined as a visual acuity of < 20/40 (vision) in both eyes, regardless of correction, as determined by the near-vision test. The length of hospital stay was calculated from the date of admission to discharge.
